# Infliximab-Induced Pulmonary Sarcoidosis Treated With Upadacitinib: A Case Report and Review of the Literature

**DOI:** 10.7759/cureus.82002

**Published:** 2025-04-10

**Authors:** Ei Swe, Jakob Begun

**Affiliations:** 1 Gastroenterology, Mater Hospital Brisbane, Brisbane, AUS

**Keywords:** anti-tnf, inflammatory bowel disease, infliximab, sarcoidosis, upadacitinib

## Abstract

Sarcoidosis is a multisystem granulomatous disorder of unclear aetiology. It can develop de novo or occur as a reaction to certain medications. While infliximab is commonly used for treating inflammatory conditions such as inflammatory bowel disease, paradoxical sarcoidosis secondary to tumor necrosis factor alpha (TNF-α) inhibitors is a rare complication. There is emerging evidence to support the use of Janus kinase (JAK) inhibitors in refractory sarcoidosis. Here, we present the first reported case of infliximab-induced pulmonary sarcoidosis in a patient with Crohn's disease successfully treated with upadacitinib and review the literature.

## Introduction

Sarcoidosis is a systemic inflammatory disorder characterized by the formation of non-caseating granulomas, which are organized clusters of immune cells that do not contain necrosis. It typically presents with bilateral hilar lymphadenopathy and reticular opacities in the lungs and can also involve the skin, eyes, and joints. The pathogenesis of sarcoidosis involves a complex interplay of genetic predisposition and environmental triggers, with T cells playing a crucial role in driving the immune response [1]. The T-cell infiltrate is characterized by an inverted CD4/CD8 ratio and elevated levels of pro-inflammatory cytokines, including tumor necrosis factor alpha (TNF-α), interleukin 2 (IL-2), and interferon-gamma (IFN-γ), which contribute to granuloma formation through macrophage activation and aggregation [1].

Crohn's disease is a chronic inflammatory bowel disorder characterized by transmural inflammation that can affect any segment of the gastrointestinal tract. Although Crohn's disease primarily affects the gut, it is also associated with extraintestinal manifestations, including granulomatous inflammation in the lungs, resembling sarcoidosis. The co-existence of Crohn's disease and sarcoidosis in a single patient is a rare phenomenon that was first documented in 1947, with only a handful of definitive cases reported in the literature. Notably, researchers such as Schürmann et al. and Willoughby et al. have proposed a potential genetic link between these two conditions [2-4]. However, the connection between these conditions and the underlying genetic mechanism remains to be fully elucidated.

Anti-TNF therapies, such as infliximab (IFX) and adalimumab (ADA), are effective treatments for patients with multiple systemic inflammatory disorders, such as inflammatory bowel disease, autoimmune arthropathies, and sarcoidosis, as they inhibit the pro-inflammatory cytokine TNF-α, reducing inflammation and promoting remission. They are also associated with an increased risk of tuberculosis (TB) reactivation, particularly in endemic regions, and screening for latent TB is important prior to initiating anti-TNF therapies.

Interestingly, anti-TNF therapies have paradoxically been linked to the development or exacerbation of inflammatory conditions, including sarcoidosis. This may be due to the disruption of normal TNF-α signaling, which is essential for regulating immune responses and preventing inappropriate granulomatous inflammation. The activation of alternative immune pathways in response to anti-TNF agents can lead to the onset of sarcoidosis in predisposed individuals [5]. The need to distinguish between sarcoidosis and TB is crucial in clinical practice, as both conditions can present with similar radiological and histopathological findings, necessitating careful diagnostic evaluation.

These paradoxical reactions of anti-TNF therapies are usually managed by cessation of the medication, together with the use of systemic corticosteroids or other anti-inflammatory agents [6]. Janus kinase (JAK) inhibitors are used in a variety of immune-mediated inflammatory disorders, including inflammatory bowel disease. Recently, JAK inhibitors have emerged as a potential treatment option for sarcoidosis, although there is limited evidence of their effectiveness in this context [7]. We present a unique case of infliximab-induced pulmonary sarcoidosis in a patient with Crohn's disease that was successfully treated with upadacitinib.

## Case presentation

A 32-year-old white woman presented to the emergency department (ED) with sudden-onset pleuritic chest pain accompanied by a dry cough. Two days prior to her presentation, she developed a 2-3 cm tender erythematous nodule on the medial aspect of her right leg. A Doppler ultrasound ruled out deep vein thrombosis. She denied any fever, nausea, vomiting, or diarrhea. She had a history of ileocolonic Crohn's disease (Montreal Classification A2L3B2), diagnosed 18 months prior to this presentation. Her initial colonoscopy revealed pancolitis and ileitis with a terminal ileum stricture approximately 7 cm proximal to the ileocecal valve. Infliximab was initiated 16 months prior to current ED presentation, and she achieved clinical remission as well as endoscopic remission six months after the introduction of infliximab 5 mg/kg every eight weeks, in combination with azathioprine 100 mg daily. She had no history of smoking, alcohol use, or familial autoimmune diseases.

On examination, the blood pressure was 110/60 mmHg, heart rate 80 beats per minute, respiratory rate 18 breaths per minute, oxygen saturation 98% on room air, and temperature 37°C. Cardiovascular and respiratory examinations were unremarkable, and there was no palpable lymphadenopathy. An electrocardiogram showed normal sinus rhythm. Laboratory investigations revealed normal blood counts, electrolytes, renal function, liver function tests, and a mildly elevated C-reactive protein of 15. Her troponin level and brain natriuretic peptide were normal. The QuantiFERON-TB Gold test was negative.

**Figure 1:**
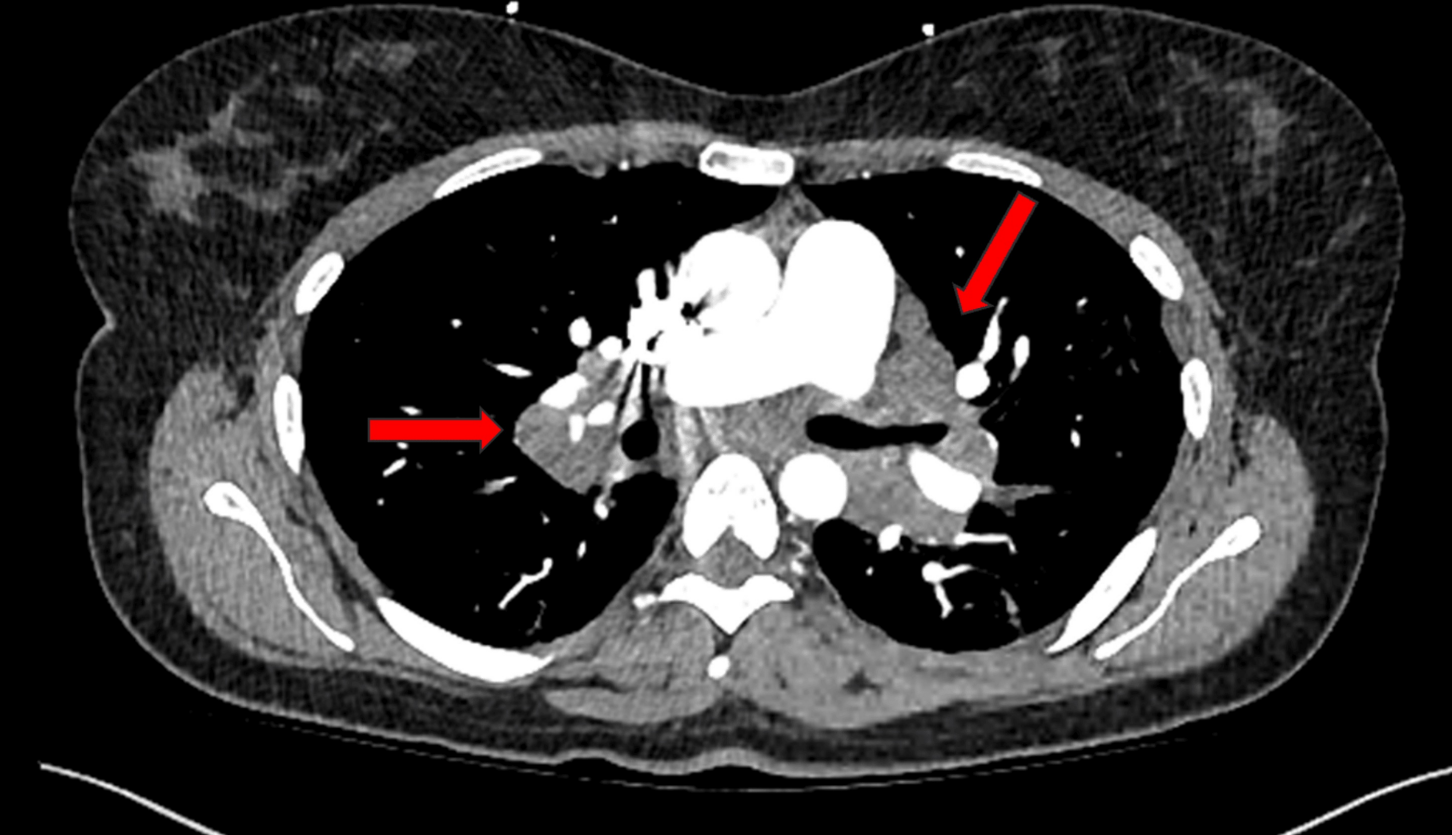
CT of the chest showing enlarged mediastinal and hilar lymph nodes CT: computed tomography

A chest X-ray showed patchy opacities in the upper lung zones. Subsequent computed tomography (CT) of the chest revealed bulky hilar and mediastinal lymphadenopathy with extensive pulmonary nodules in the mid to upper lung zones, highly suggestive of sarcoidosis (Figure 1). She received intravenous antibiotics without improvement in symptoms.

**Figure 2:**
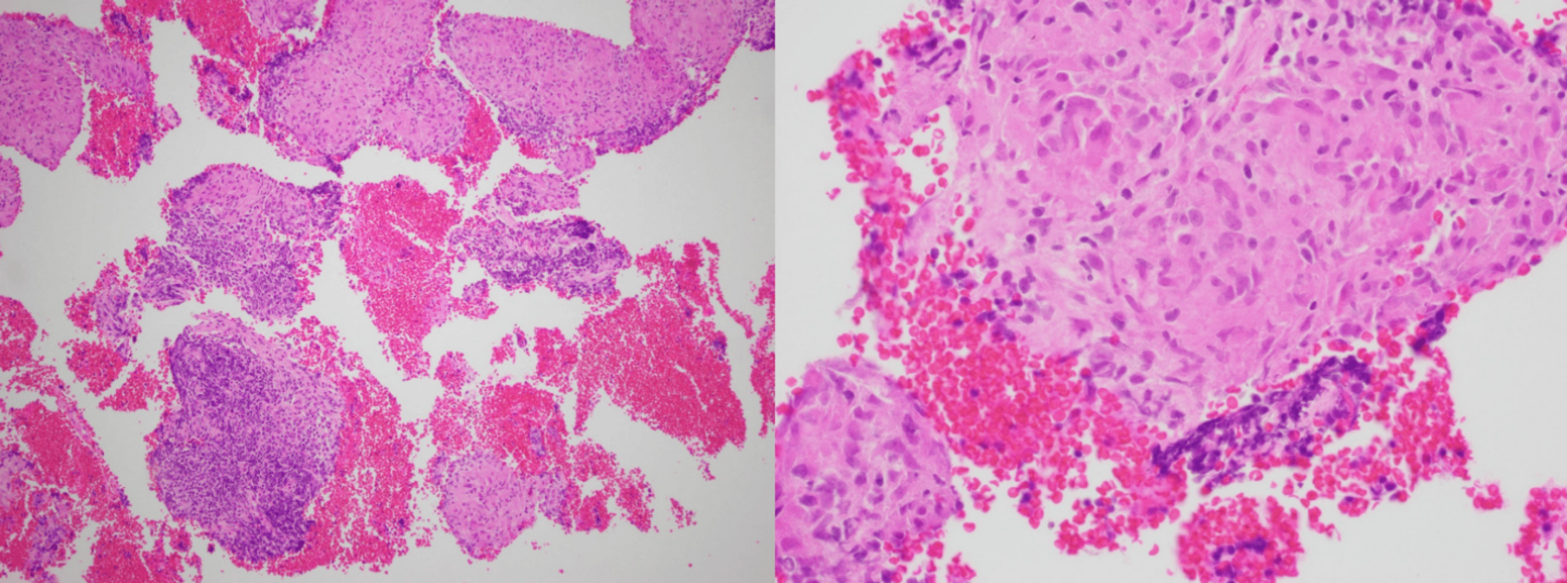
Non-caseating granulomas in bronchial biopsies

Due to the radiological findings, a bronchoscopy was performed, revealing cobblestone nodularity of the bronchial mucosa with granulomatous changes. Endobronchial ultrasound (EBUS) demonstrated homogenous, triangular lymph nodes, and biopsies confirmed non-caseating granulomas consistent with sarcoidosis (Figure 2). The findings were atypical for TB or Crohn's disease involvement, with negative acid-fast bacillus staining and mycobacterial cultures. Cytology was negative for malignancy.

**Figure 3:**
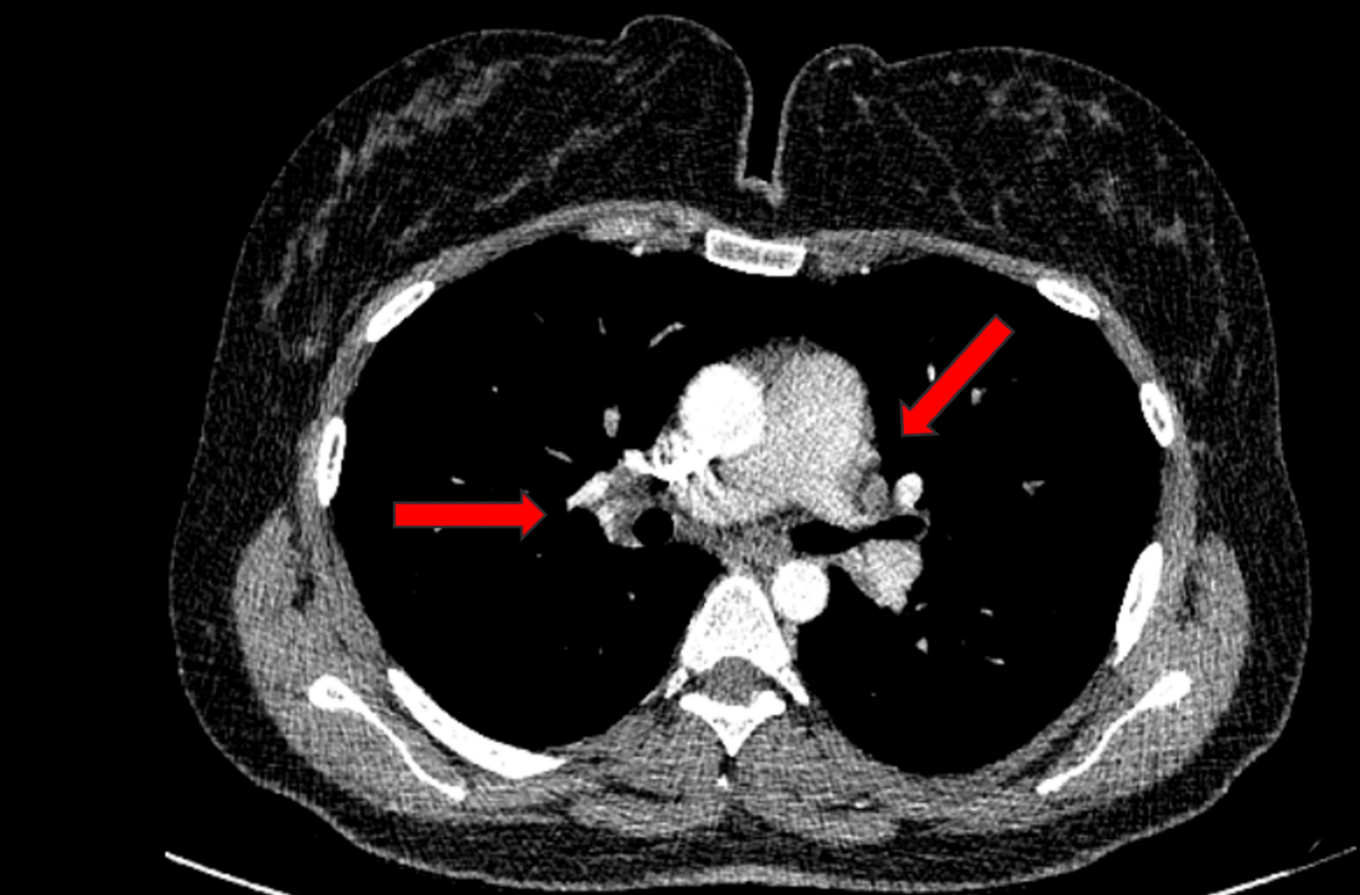
Repeat CT of the chest 11 weeks post-initiation of upadacitinib showing the near-resolution of hilar lymphadenopathy CT: computed tomography

Given her medication history, infliximab-induced sarcoidosis was suspected. Infliximab was discontinued, and she was commenced on upadacitinib 45 mg. After eight weeks, her pulmonary symptoms, including cough, improved significantly. Her Crohn's disease remained in clinical remission. A follow-up CT of the chest after 11 weeks demonstrated a near-complete resolution of the mediastinal lymphadenopathy and pulmonary nodules (Figure 3). After 12 weeks of upadacitinib 45 mg, the upadacitinib dose was reduced to 30 mg daily as a maintenance dose. As she remained free of recurrent respiratory symptoms on upadacitinib 30 mg for three months, and the most recent CT of the chest showed a near-complete resolution of mediastinal lymphadenopathy, further imaging was deemed unnecessary at this stage.

## Discussion

The relationship between TNF-α inhibitors, such as infliximab, and the onset of sarcoidosis is a topic of ongoing interest. While TNF-α inhibitors are widely used to treat multiple inflammatory conditions, including inflammatory bowel disease and inflammatory spondyloarthropathy, there is a growing body of literature documenting paradoxical reactions, such as drug-induced sarcoidosis. The exact mechanisms underlying this phenomenon remain unclear; however, it is hypothesized that TNF-α inhibition disrupts the maintenance of granulomas, leading to immune dysregulation and the subsequent development of granulomatous inflammation. This immune imbalance could potentially activate alternative pathways that promote the onset of sarcoidosis in genetically predisposed individuals [5].

Several case reports and case series in the literature document instances of TNF-α inhibitor-induced sarcoidosis. Most cases have been reported in patients with underlying inflammatory arthropathies. To our knowledge, there have been 10 reported cases of anti-TNF inhibitor-induced sarcoidosis in patients with inflammatory bowel disease, as summarized in Table 1 [8-16]. Of these, eight patients had Crohn's disease, and two had ulcerative colitis. The duration of exposure to anti-TNF ranged from seven months to five years with six patients on infliximab and four on adalimumab. TNF-α inhibitors were ceased in most cases (eight patients), and five patients were treated with systemic corticosteroids [8-16]. One patient with cutaneous sarcoidosis continued infliximab with pre-dosed oral diphenhydramine 25 mg and intravenous hydrocortisone 200 mg [9].

**Table 1:** Anti-TNF-induced sarcoidosis in patients with underlying inflammatory bowel disease TNF: tumor necrosis alpha; CD: Crohn's disease; UC: ulcerative colitis; M: male; F: female; IFX: infliximab; IV: intravenous; ADA: adalimumab; AZA: azathioprine; m: months; y: years

Reference	Year published	Disease	Age/sex	Anti-TNF/duration	Organs involved	Treatment and outcome
[8]	2010	CD	35/M	IFX/7m	Skin, pulmonary bilateral nodular infiltrates	Ceased IFX. No other medication. Symptoms improved
[9]	2012	UC	66/F	IFX/23m	Skin	Continued IFX, with pre-dosed diphenhydramine 25 mg oral and IV hydrocortisone 200 mg
[10]	2013	CD	25/F	ADA/18m	Pulmonary, spleen nodules	AZA ceased. 8 months of steroid. ADA continued as monotherapy
[11]	2014	CD	37/M	ADA/2y	Skin, pulmonary	ADA ceased
[12]	2015	CD	44/F	IFX/5y	Spinal cord	Ceased IFX. High-dose steroid
[13]	2017	CD	30/M	IFX/18m	Pulmonary	Resolved lung nodules 5 m after IFX ceased
[14]	2017 (2 cases)	CD	30/M	ADA/5y	Uveitis/bilateral mediastinal and hilar adenopathies	Oral methyl prednisolone
		CD	21/M	ADA/18m	Mediastinal and hilar adenopathies. Erythema nodosum	Oral methyl prednisolone
[15]	2017	UC	30/M	IFX/3.5y	Pulmonary sarcoidosis	Ceased IFX. Treated with corticosteroid
[16]	2021	CD	37/F	IFX/15y	Bilateral pulmonary nodules and hilar LNs	Ceased IFX. Symptoms resolved 4 months after

The management of sarcoidosis is contingent upon the severity of the clinical presentation and the specific organs involved. According to current clinical guidelines, the standard approach to treating sarcoidosis typically involves the administration of corticosteroids as the first-line therapy. In cases where corticosteroids alone are insufficient, immunomodulatory agents such as azathioprine and methotrexate may be considered adjunctive treatments. For patients with refractory disease, the use of biologic therapies, including infliximab, adalimumab, and rituximab, may be warranted to achieve better disease control [17-19].

JAK inhibitors target the JAK/STAT signaling pathway, which plays a critical role in regulating inflammation, immune cell function, and granuloma formation in sarcoidosis. Dysregulation of this pathway, particularly through cytokines like IFN-γ, IL-2, and IL-23, is implicated in the pathogenesis of sarcoidosis, with evidence showing increased JAK/STAT activity in affected tissues [20]. Clinical studies and several case reports have demonstrated that JAK inhibitors, such as tofacitinib, ruxolitinib, and baricitinib, can effectively reduce inflammatory markers, improve clinical outcomes, and provide disease control in patients with refractory sarcoidosis, particularly those with cutaneous or pulmonary involvement [7,21-23]. A recent review by Talty et al. summarized the use of JAK inhibitors in sarcoidosis, reporting 21 patients treated with these agents, including 15 patients treated with oral tofacitinib and three patients with oral ruxolitinib, further highlighting their therapeutic potential [7]. These therapies work by selectively inhibiting specific JAKs, thereby modulating the activity of key cytokines involved in the disease [20]. Upadacitinib, a selective JAK1 inhibitor, has shown promise in treating inflammatory bowel disease, and there is growing evidence supporting its efficacy in sarcoidosis. Our case report serves as the first documented case of upadacitinib effectively treating infliximab-induced pulmonary sarcoidosis in a patient with Crohn's disease. 

This case highlights the importance of recognizing infliximab-induced sarcoidosis in the differential of patients presenting with respiratory symptoms and offers insight into the potential therapeutic role of upadacitinib, especially in patients with concomitant immune-mediated inflammatory diseases.

## Conclusions

This case highlights the occurrence of infliximab-induced pulmonary sarcoidosis, a rare but significant complication that healthcare providers should remain vigilant for, especially in patients presenting with respiratory symptoms while receiving anti-TNF therapy, especially after ruling out TB, which is an important differential diagnosis given the increased risk of reactivation with anti-TNF therapy. The successful treatment of infliximab-induced pulmonary sarcoidosis with upadacitinib demonstrates its potential as a viable therapeutic option. Further research is required to explore the efficacy and safety of JAK inhibitors in managing drug-induced complications such as sarcoidosis. 
